# Antipsychotic Use in Pregnancy: Patient Mental Health Challenges, Teratogenicity, Pregnancy Complications, and Postnatal Risks

**DOI:** 10.3390/neurolint14010005

**Published:** 2022-01-03

**Authors:** Amber N. Edinoff, Niroshan Sathivadivel, Shawn E. McNeil, Austin I. Ly, Jaeyeon Kweon, Neil Kelkar, Elyse M. Cornett, Adam M. Kaye, Alan D. Kaye

**Affiliations:** 1 Department of Psychiatry and Behavioral Medicine, Louisiana State University Health Science Center Shreveport, Shreveport, LA 71103, USA; Niroshan.Sathivadivel@lsuhs.edu (N.S.); shawn.mcneil@lsuhs.edu (S.E.M.); 2 College of Medicine, The University of Tennessee Health Science Center, Memphis, TN 38103, USA; aly1@uthsc.edu; 3 School of Medicine, Louisiana State University Health New Orleans, New Orleans, LA 70112, USA; jkweon@lsuhsc.edu; 4 College of Medicine-Phoenix, University of Arizona, Phoenix, AZ 85004, USA; nkelkar@email.arizona.edu; 5 Department of Anesthesiology, Louisiana State University Health Science Center Shreveport, Shreveport, LA 71103, USA; elyse.bradley@lsuhs.edu (E.M.C.); alan.kaye@lsuhs.edu (A.D.K.); 6 Department of Pharmacy Practice, Thomas J. Long School of Pharmacy and Health Sciences, University of the Pacific, Stockton, CA 95211, USA; akaye@PACIFIC.EDU

**Keywords:** antipsychotics, pregnancy, teratogenicity, complications, psychosis

## Abstract

Pregnant women constitute a vulnerable population, with 25.3% of pregnant women classified as suffering from a psychiatric disorder. Since childbearing age typically aligns with the onset of mental health disorders, it is of utmost importance to consider the effects that antipsychotic drugs have on pregnant women and their developing fetus. However, the induction of pharmacological treatment during pregnancy may pose significant risks to the developing fetus. Antipsychotics are typically introduced when the nonpharmacologic approaches fail to produce desired effects or when the risks outweigh the benefits from continuing without treatment or the risks from exposing the fetus to medication. Early studies of pregnant women with schizophrenia showed an increase in perinatal malformations and deaths among their newborns. Similar to schizophrenia, women with bipolar disorder have an increased risk of relapse in antepartum and postpartum periods. It is known that antipsychotic medications can readily cross the placenta, and exposure to antipsychotic medication during pregnancy is associated with potential teratogenicity. Potential risks associated with antipsychotic use in pregnant women include congenital abnormalities, preterm birth, and metabolic disturbance, which could potentially lead to abnormal fetal growth. The complex decision-making process for treating psychosis in pregnant women must evaluate the risks and benefits of antipsychotic drugs.

## 1. Introduction

Mental health disorders represent 7% of the global burden of disease [1]; however, the topic remains overlooked and, frequently, misjudged in society [2]. Recent WHO guidelines continue to emphasize its significance by highlighting the association between mental health disorders and poorer health outcomes [3]. A particularly vulnerable population includes pregnant women, with 25.3% of pregnant women classified as suffering from a psychiatric disorder [4].

Of pregnant women with a psychiatric disorder between 2006 and 2011, around 6% to 15% were prescribed antipsychotic medication [5]. Since childbearing age typically aligns with the onset of mental health disorders, it is of utmost importance to consider the effects that antipsychotic drugs have on the developing fetus. Conditions such as schizophrenia, bipolar disorder, and depression may require pharmacological intervention to manage the symptoms and to keep the patient and her fetus safe. However, the induction of pharmacological treatment during pregnancy may pose significant risks to the developing fetus.

Avoiding the risks of antipsychotics may require simply asking patients to halt pharmacological agents altogether. However, it is essential to consider the risk of leaving pregnant women untreated. Recent reports suggested that maternal psychiatric disorders may reduce cognitive function and result in impaired brain development in the fetus [6]. An increased risk of self-harm, substance abuse, and inadequate prenatal care may complicate the care plans moving forward [7]. For untreated pregnant women with bipolar disorder, relapse rates were cited as high as 71% [8]. The emergence of psychosis during pregnancy is an emergency to both the mother and the fetus, leading to exceptionally poor cooperation and care during delivery [9,10]. Discontinuing medication is typically reserved for patients with a history of mild psychiatric illness. Psychosocial therapies and other nonpharmacologic treatments generally are implemented to manage the symptoms [11,12]. Antipsychotics are typically introduced when the nonpharmacologic approaches fail to produce desired effects or when the risks outweigh the benefits from continuing without treatment or the risks from exposing the fetus to medication.

While antipsychotic drugs are especially beneficial for managing acute manifestations of psychoses, the side effects are troubling and may lead to discontinuing the pharmacological agents [13]. Adherence issues are not uncommon [14], which is a significant risk factor for relapse [15]. Risk is typically assessed based on adverse pregnancy outcomes and long-term neurobehavioral deficits. Unfortunately, the research behind the associated risks for antipsychotic treatment is not entirely understood. Many studies have demonstrated an association between fetal exposure to antipsychotics during pregnancy with preterm birth, congenital malformations, and abnormally slow fetal growth [16,17,18]. On the other hand, a separate study could not confirm the associations, especially with the delayed fetal growth [19].

Therefore, this review aims to evaluate (1) Antepartum psychiatric disorders, (2) Antipsychotics overview/classes, (3) Antipsychotics in pregnancy, and (4) Clinical studies to critically assess the teratogenicity, pregnancy complications, and postnatal risks associated with fetal exposure to antipsychotic treatment during pregnancy.

## 2. Antepartum Psychiatric Disorders

Historically, pregnant women have always been considered a high-risk population. According to the World Health Organization, 10% of antepartum and 13% of postpartum patients have experienced a psychiatric disorder worldwide, with an increasing trend in developing countries [20]. Antepartum psychiatric disorders (APDs) are a significant cause of disability among women in the perinatal period. It is vital to diagnose and treat APDs properly because they may have negative consequences for the growth and development of the fetus [21]. Perinatal mortality rate, congenital malformations, low Apgar scores, and low birth weight were significantly increased in women with APDs [22]. Furthermore, depression during pregnancy can increase the risk of postpartum depression and can impede mother–child attachment, caregiving, and child growth and development [23]. Are pregnant women more susceptible to psychiatric disorders? Although the incidence of major depressive disorder may be increased during the postpartum period, the incidence rates of a psychiatric disorder were almost equivalent among antepartum women, postpartum women, and non-pregnant women [4]. However, the associated risks of APDs in general seem to be more correlated with low socioeconomic status, younger age, HIV infection, and intimate partner violence during pregnancy [24,25,26]. According to DSM-IV criteria, the most prevalent antepartum psychiatric disorders were major depressive disorder (MDD), anxiety disorder, and psychosis in antepartum women.

While sadness and grief are normal responses to pregnancy complications, depression may lead to a disabling psychiatric disorder, such as major depressive disorder or bipolar disorder, that increases the risk of unfavorable obstetric outcomes. If untreated, antepartum depression may worsen into other types of psychiatric illness, and in a worst-case scenario, may lead to maternal suicide attempts. Antepartum women with comorbid depression and anxiety also produce excessive amounts of cortisol that further increase the already heightened cortisol levels during normal pregnancies, and elevated cortisol levels with a severe depressive disorder may increase the risk of complications during pregnancy [27].

Psychosis is a general term to describe a psychiatric disorder with delusions, disorganized thinking and speech, hallucinations, and/or other associated behavioral and cognitive impairments interfering with the ability to meet the ordinary demands of life [28]. Psychosis occurs with other psychiatric disorders, including, but not limited to, schizophrenia, schizoaffective disorders, bipolar disorders, and major depressive disorders. Similar to APDs, pregnant women with psychosis have elevated risks of several adverse obstetric and neonatal outcomes [29].

Schizophrenia is a collection of signs and symptoms of unknown etiology, primarily defined by observed signs of psychosis. Women with schizophrenia are at a higher risk for relapse during and after pregnancy [30]. Early studies of pregnant women with schizophrenia showed an increase in perinatal malformations and deaths among their newborns [31,32]. Furthermore, studies have shown that schizophrenia is associated with an increased relative risk for pre-eclampsia, venous thromboembolism [33,34,35], and compared to women with no psychiatric disorder, infants had increased risks for preterm birth, low birth weight, lower Apgar score, and fetal or neonatal deaths [33,34,36].

Bipolar disorder (BD) is a severe mood disorder characterized by a wide range of mood changes between depressive, hypomanic, manic, or mixed episodes. Undiagnosed BD is associated with a higher risk of suicide, higher socio-occupational dysfunction rates, and increased economic burden. BD increases the risk of postpartum psychosis in pregnant women, which is associated with obstetrics suicide or infanticide [37]. A study done by Vesga-López shows that there is no difference in the prevalence of BD in pregnant women when compared with non-pregnant women in the US [38]; but, similar to schizophrenia, women with BD have an increased risk of relapse in antepartum and postpartum periods [39]. In addition, babies of women with antepartum BD may have increased risks for congenital abnormalities, low birth weight, neonatal readmissions, and neonatal morbidity [40,41].

Borderline Personality Disorder (BPD) is a personality disorder that has a female predominance. This is a disorder that is characterized by emotional liability, unstable relationships, impulsivity, and sometimes is associated with self-harm. BPD is associated with a high risk of suicide, which is linked to the impulsivity seen in the disorder [42]. Although medical treatment is usually reserved for co-occurring psychiatric disorders, sometimes second-generation antipsychotics can be used to help treat the impulsivity that can lead to self-harm. Aripiprazole, olanzapine, risperidone, and ziprasidone are second-generation antipsychotics that have shown some efficacy in decrease the impulsivity seen in BPD [43]. Suicidality seen in BPD is better treated with psychotherapy interventions, such as dialectical behavioral therapy [44].

## 3. Antipsychotics Overview/Classes

Antipsychotic drugs are a class of drugs used to reduce psychotic symptoms in patients with psychiatric disorders. Bipolar disorder and schizophrenia are commonly treated with antipsychotics, but different variants of psychoses may also receive similar treatment, such as dementia-related psychosis, depression with psychotic features, and drug-induced psychoses. Antipsychotics are also available off-label for various disorders, including sleep disorders, obsessive-compulsive disorder, augmentation in depression, and dementia. In this regard, augmentation of depression with some antipsychotics is “on label” for select antipsychotics.

### 3.1. Classification

The classification scheme employing “typical” and “atypical” is the preferred nomenclature based on the liability to cause EPS. However, this should not be confused with other similar classification schemes such as “first” and “second” generation or “chlorpromazine-like” and “clozapine and related drugs.” The first and second-generation terminology describes the drugs discovered before and after clozapine, respectively. Clozapine and related drugs provide a more potent blockade of the 5HT_2_ serotonin receptor subtype, 5-HT_2A_, instead of the powerful D_2_-receptor blockade characteristic of chlorpromazine-like drugs.

### 3.2. Receptor Binding

The serotonin hypothesis of schizophrenia started with discovering that LSD and mescaline were agonists of the serotonin (5-HT) receptor. Identifying various 5-HT-receptor subtypes led to finding the vital mediator for hallucination effects and, more importantly, the basis for the antipsychotic agents: the 5-HT_2A_-receptor antagonist in addition to dopamine receptor blockade. Blockade of this receptor is key to the atypical group of antipsychotics, of which clozapine represents the prototypical drug. 5-HT_2A_-receptors in the cortex, limbic region, and striatum modulate the release of several neurotransmitters such as dopamine, GABA, glutamate, and acetylcholine. Furthermore, stimulating the 5-HT_2A_-receptor depolarizes the glutamate neurons and stabilizes the N-methyl-D-aspartate (NMDA) receptors on post-synaptic neurons [45,46,47].

Serotonin’s role in schizophrenia receives less attention than the dopamine hypothesis of schizophrenia, but it should be discussed, especially when considering the positive and negative symptoms of schizophrenia. The dopamine receptors have important implications when assessing the antipsychotic mechanisms of action: many antipsychotics inhibit the postsynaptic D_2_-receptors in the mesolimbic and striatal-frontal pathways. Additionally, it was found that dopamine receptor agonists aggravate symptoms in schizophrenia patients. Greater dopamine levels and increased density of dopamine receptors, specifically, D_2_-receptors in the nucleus accumbens, caudate, and putamen, were discovered in patients with schizophrenia. The D_2_-receptors provide G_i_-coupled inhibition of adenylyl cyclase to decrease cAMP. Calcium channels are inhibited while potassium channels remain open. The typical antipsychotics are known to produce antipsychotic effects with more selective D_2_-receptor block and typically result in EPS when more than 80% of the D_2_-receptors are occupied. However, despite the several lines of evidence associating dopamine’s role in schizophrenia, the reduced dopamine activity found in newer antipsychotics has created a shift in the pharmacologic approach towards manipulating other receptors such as the 5-HT_2A_-receptor subtype [47,48].

### 3.3. Atypical Antipsychotic Drugs

The atypical antipsychotics include olanzapine, quetiapine, iloperidone, lurasidone, paliperidone, risperidone, and ziprasidone. These drugs are grouped based on a similar mechanism of action: more potent 5-HT_2A_-receptor antagonism than D_2_-receptor antagonism. These drugs are inverse agonists of the 5-HT_2A_-receptor in which inhibit the constitutive activity of the receptors. Most are also 5-HT_1A_ partial agonists and either 5-HT_6_ or 5-HT_7_-receptor antagonists. 5-HT_1A_ partial agonism creates a synergistic effect with the 5-HT_2A_-receptor antagonism [46,47]. Aripiprazole has a slightly different mechanism of action: it reduces dopaminergic neurotransmission as a partial D_2_-receptor agonist [49].

A smaller group of antipsychotics is worth mentioning as these drugs have a mechanism of action that differs slightly from the larger group of atypical antipsychotics. It is suggested that amisulpride and cariprazine have greater D_2_/D_3_-receptor antagonism with a similar serotonergic profile to the clozapine-like drugs. Amisulpride is a potent 5-HT_7_-receptor antagonist, whereas cariprazine is a 5-HT_2B_-receptor antagonist and a 5-HT_1A_-receptor partial agonist [50,51].

## 4. Antipsychotics in Pregnancy

Antipsychotic drugs are often prescribed to patients as the standard of care for bipolar disorder, schizophrenia, and other psychotic disorders. They are also prescribed to a lesser degree for depression, anxiety, insomnia, autism, and nausea in early pregnancy [52,53]. Over several decades, the availability of effective treatment for psychotic patients has led to an overall increase in wellness and fertility rates among women with psychosis; however, pregnancy complicates antipsychotic treatment options. Whether or not prescribing antipsychotic drugs to antepartum women would be beneficial is a challenging dilemma. Treating the mother with antipsychotics implies exposing the fetus to the drug, potentially harming the patient’s child. It is known that antipsychotic medications can readily cross the placenta [54], and exposure to antipsychotic medication during pregnancy is associated with potential teratogenicity. Potential risks associated with antipsychotic use in pregnant women include congenital abnormalities [55], preterm birth [16], and metabolic disturbances [19], which could potentially lead to abnormal fetal growth. On the other hand, abstaining from antipsychotics may result in a worsened prognosis due to the deteriorated psychiatric condition of a mother, which is a more significant threat to the mother and child [29]. Furthermore, discontinuation of antipsychotic treatment during pregnancy may increase the risk of relapse of psychiatric disorders, including bipolar disorder and schizophrenia [8]. Thus, clinicians are often faced with the challenge of balancing the benefits and potential risks of antipsychotic use during pregnancy.

Changes in physiology during pregnancy also result in changes in the pharmacokinetics of multiple medications, including antipsychotics. For instance, an increased dose of antipsychotic medication may be required to achieve the same serum concentration of the antipsychotic during pregnancy because of the isozymes of the P450 enzyme system or increased blood flow and increased renal elimination of these drugs due to increased glomerular filtration rate during pregnancy [56,57].

The use of antipsychotics during pregnancy has obstetric implications. The major nonpsychiatric maternal health complications are the development of diabetes and weight gain, especially in second-generation antipsychotics (SGAs), some of which are known to increase the risk of diabetes mellitus in general adult patients [58]. While examining both atypical and typical antipsychotics during pregnancy, patients were observed with a nearly twofold increase in the gestational diabetes mellitus risk in women [58]. Additionally, maternal antipsychotic medication (both FGAs and SGAs) may be associated with low birth weight, cesarean delivery, or elevated risk for prematurity [58].

One of the biggest concerns regarding the use of any antipsychotics in pregnancy is the risk of teratogenicity to the fetus, especially during the first trimester, when it is the most critical period for organ formation. However, it remains unclear to what extent antipsychotics cause complications in the neonatal period. Most exposure to antipsychotics is unavoidably coupled with maternal psychiatric disorders and associated comorbidities such as malnutrition, smoking, substance abuse, alcohol abuse, physical illnesses, and traumas [55]. Thus, it is challenging to eliminate confounding variables to isolate the specific influence of antipsychotic medication on fetal outcomes. Finally, because conducting randomized controlled trials in pregnant women is unethical, there is a lack of high-quality evidence for the risks of antipsychotics in pregnancy.

## 5. Clinical Studies

Older drugs such as haloperidol or chlorpromazine have extensive studies assessing their effects during pregnancy and breastfeeding [59]. In contrast, newer atypical antipsychotics have limited well-controlled human studies, yet show teratogenic effects in all atypical pharmaceuticals in animal studies [60]. Animal models show evidence of skeletal malformations, central nervous system (CNS) defects, cleft palate, cardiac abnormalities, decreased fetal growth, and fetal death [61,62,63].

Most studies of antipsychotics in this patient group are primarily interested in the developing fetus. However, some studies focus on the effects on women during pregnancy. A Swedish study examined mothers from 1 July 2005 to 31 December 2009 who were on olanzapine/clozapine (*n* = 169) or other antipsychotics (*n* = 338) and assessed the effects of these medications to those who were not on any antipsychotics (*n* = 357,696). Results showed an increased risk (OR 1.77; 95% CI 1.04–3.03) of patients developing gestational diabetes. The study indicated that those who used antipsychotics had increased chances of developing gestational diabetes. The study also reported infants being small for gestational age, but attributed it to confounding factors (e.g., tobacco use during pregnancy) rather than a pharmacological cause [19]. The increased risk of gestational diabetes additionally places a known risk of gestational diabetes complications on the fetus [64].

There are known complications during labor. From 2005 to 2012, 147 mothers who were taking an antipsychotic were followed through pregnancy to 1 year after birth. Of the 147, there were 142 live births, with 25 (18%) being pre-term births [65]. Higher doses of antipsychotics were correlated with the likelihood of pre-term birth. Additional complications noted were 56 (43%) of babies required special care nursery or intensive care after birth, 48 (37%) had some form of respiratory distress, 20 (15%) showed signs of withdrawal, and 8 (6%) had congenital abnormalities [65]. The rates were compared to the expected rate to examine any significant differences [65,66]. Although the study shows gestation and early life complications, most pregnancies had the birth of healthy babies [65]. Clinicians must balance the mother’s clinical characteristics and consider the positive long-term complications of the vast number of pregnancies.

In 2020, a study in Finland assessed 20 years of pregnancy (1996–2016) to compare 1,181,090 pregnancies [67]. Women were separated into three groups: exposed to FGA (*n* = 1576), exposed to SGs (*n* = 4115), and no antipsychotic exposure (*n* = 22,125). The SGA pregnancy outcomes were compared to the other two comparison groups to assess any statistically significant differences. When compared to the unexposed group, SGAs were noted to have an increased risk of maternal diabetes (OR 1.43; 95% CI 1.25–1.65), rates of cesarean section (OR 1.35; 95% CI 1.18–1.53), and preterm birth (OR 1.29; 95% CI 1.03–1.62). Additionally, the study reported an increase in infants being born large for gestational age (LGA) when compared to both unexposed (OR 1.57; 95% CI 1.14–2.16) and FGA exposed (OR 1.89, 95% CI 1.20–2.99) groups [67]. This study also helps support the hypothesis that prenatal exposure to SGA is involved with an increased risk of pregnancy complications related to the impairment of glucose metabolism.

Other effects have been observed for FGA. One study examined women taking FGAs during their third trimester of pregnancy (*n* = 29) and compared them to an unexposed cohort (*n* = 29). The infants were assessed at 3 and 14 days of age using the Brazelton Neonatal Behavioral Assessment scale to gauge neurophysiological function. On day 3 of life, there were no significant differences between the cohorts (*p* = 0.08), but on day 14, there was a statistically significant effect due to autonomic instability (*p* = 0.04). The result revealed that mothers taking FGA during their third trimester can put the neonate at risk for extrapyramidal symptoms (EPS), possibly as a form of neonatal abstinence or withdrawal [68].

Animal studies of mothers taking antipsychotics have resulted in congenital malformations. However, studies with these malformations have significantly higher doses than those recommended for human therapeutic doses [61,69,70]. One study followed 561 pregnant women exposed to SGA and compared them to 284 pregnant women exposed to FGA and 1122 unexposed women. Major malformation rates in the SGA cohort were higher than the unexposed cohort (OR: 2.17; 95% CI 1.20–3.91). In this regard, the study reported that mothers in the unexposed cohort and SGA and FGA cohorts had many confounding factors (e.g., alcohol use, smoking, BMI, unplanned pregnancy, etc.). The study also attributed a detection bias for atrial septal defects and ventricular septal defects due to not distinguishing between minor and major malformations [71]. Other studies examining SGA polytherapy also have reported an increase in malformations in the SGA-exposed cohort but did not have enough statistical power for it to be statistically significant [72].

However, other studies examining the reproductive safety of antipsychotics report no significant difference. A study conducted at Massachusetts General Hospital examined the risks of major malformations in mothers taking SGA during pregnancy and compared it to unexposed mothers. Three hundred and three mothers were enrolled in the study, with 214 live births with first-trimester exposure to an SGA and 89 mothers as the control. The experimental group had three major malformations, and the control group had one major malformation (OR 1.25; 95% CI 0.13–12.19). Their results show that it is unlikely for SGA to increase the risk of major malformations more than 10 times that of the general population [73]. The study does highlight some limits to the methodology of these cohort studies, such as the generalizability of the study, the statistical power, and balancing the management of the mother’s physical and mental wellbeing.

Another study addressed butyrophenone neuroleptics (haloperidol and penfluridol) safety during pregnancies from 1989 to 2001. Mothers exposed to haloperidol (*n* = 188) and penfluridol (*n* = 27) during their first trimester were compared to a control cohort (*n* = 631). The study showed that the rates of congenital abnormalities did not differ between cohorts (3.4% vs. 3.8%, *p* = 0.787), but reported two cases of limb defects in the exposed groups, suggesting an emphasis on ultrasound evaluation of the limbs first-trimester exposure. Of note, the study did report a higher rate of elective abortions (8.8% vs. 3.8%, *p* = 0.004), a higher rate of preterm birth (13.0% vs. 6.9%, *p* = 0.006), a lower birth weight (3155 g vs. 3370 g, *p* < 0.001), and lower birth weight of full-term infants (3250 g vs. 3415 g, *p* = 0.004) in the butyrophenone cohorts [71]. The results demonstrated many different factors to assess when assessing safety in prescribing antipsychotics for patients during pregnancy. Should results stabilize the null hypothesis with a larger sample size, it might be reassuring for clinicians and patients when deciding about treatments during pregnancy [74].

The use of antipsychotics during lactation has been studied related to their lipophilic properties. In 2013, one study reviewing four prospective studies, 12 case series, 28 case reports, and pharmaceutical registries examined breastfeeding safety while taking antipsychotics. The study reported that few studies are based on small sample sizes and that only a few medications were studied using prospective studies. The study reported that olanzapine and quetiapine were acceptable for breastfeeding. Haloperidol, chlorpromazine, risperidone, and zuclopenthixol were considered possible for breastfeeding under medical supervision [66]. Other articles also assessed FGA and SGA use during breastfeeding to better understand their effects. Typical antipsychotics are considered safe, as they do not build up to therapeutic doses in the milk [61,67]. However, SGA medication has varying effects. Risperidone was initially believed to be unsafe for breastfeeding [61,67]. However, studies are now showing that it does not regularly affect the infant. One study examined risperidone’s active metabolite (9-hydroxyrisperidone) in two mothers’ blood and breast milk to estimate the amount the feeding infant receives. They also measured the infants’ (*n* = 2) plasma concentration to determine the amount of risperidone in the infant’s system. The milk/plasma dose for the two mothers was 2.3% and 2.8%, with no active metabolite in the infants. The study concluded that risperidone is unlikely to be hazardous to a breastfeeding individual. However, it is essential to assess risks–benefits with every patient to make sure you can balance the mother’s symptoms with the risk of antipsychotic exposure to newborns [72].

The effects of atypical antipsychotics on the fetal brain are still being assessed. One study investigated the developmental effects atypical antipsychotics have on infants born to mothers on SGAs during pregnancy. The developmental progress of infants exposed to atypical antipsychotics (*n* = 76) was compared to a matched control cohort with no exposure to any antipsychotic (*n* = 76). The study assessed several metrics, including APGAR, bodyweight, composite scores of the Bayley Scales of Infant and Toddler Development (BSID-III). At 2 months of age, the study found significantly lower scores assessing cognitive, social-emotional, motor, and adaptive behavior in the SGA-exposed cohort. Patients with more exposure to SGA in-utero had lower composite scores. At 12 months, there were no significant differences between the two groups. This suggests that fetal exposure to SGA may cause a short-term developmental delay in infants in adaptive, motor, cognitive, and social-emotional behavior, but not in language, body weight, or height. However, the delay does not last past 12 months of age. The study also suggests that women who require antipsychotic treatment should continue taking the same dose of antipsychotic medication during gestation to prevent relapse of symptoms [73].

The long-term effects of antipsychotics on the developing fetus are also factors to consider. Weight gain during antipsychotic medication is a frequent reason that patients in psychosis discontinue their medication. Several factors have been known to contribute to weight gain, but one study examined the effects of the intrauterine environment (birth weight) on patients who take antipsychotics. The study compared one cohort of antipsychotic-naive who were treated with olanzapine (*n* = 23) and one cohort of treatment-resistant patients initiated on clozapine (*n* = 24) to assess for weight gain with independent variables (birth weight, BMI, height, age, and duration of treatment). The study showed that birth weight was a significant factor in predicting weight gain in the antipsychotic naïve cohort (−7.4 +/− 3.2, *p* = 0.047). The research indicates that an early uterine environment, such as mothers taking antipsychotics, might play a role in weight change in patients who are otherwise antipsychotic naïve patients [71].

Related to the small number of samples available for analysis, it is challenging to create a proper guideline for antipsychotic management during pregnancy. It is essential to consider the mother’s medical and mental health while considering the dosages. These should also be weighed against possible exposure to the newborn, as short and long-term effects are possible. To provide a proper guideline or best practice strategy, additional prospective and retrospective data should be collected and evaluated. Table 1 summarizes the studies discussed in this section. Table 2 summarizes the possible adverse effects seen in both the mother and the fetus.

## 6. Conclusions

Mental health disorders are especially challenging to treat in pregnant women. The complex decision-making process for treating psychosis in pregnant women must evaluate the risks and benefits of antipsychotic drugs. It is possible to pause pharmacological treatment temporarily, in particular during organogenesis, e.g., typically described as days 7–57 of the first trimester of pregnancy; however, the risk of relapse and onset of debilitating symptoms may pose significant barriers to those nontreated [75]. In this regard, the option to treat may introduce substantial risks for congenital malformations and perinatal complications to the developing fetus [76]. Regardless of the treatment plan, managing the mother’s mental health status and monitoring the fetus throughout the pregnancy can ensure that appropriate measures are taken to provide more than enough care during these challenging times.

Future avenues of research should continue to unveil the safety and efficacy profile for antipsychotics since the specific interactions are not entirely understood. Current research revolves around finding atypical antipsychotic compounds or similar agents that target the mesolimbic system of the central neurotransmitter receptors to avoid side effects associated with the extrapyramidal system. Carefully designed studies should also aim to assess the impact on the developing fetus from poly-drug exposure, specifically with the inclusion of antipsychotics. With genetic research growing exponentially in recent years, analysis of genetic and environmental contributions to antipsychotic drug efficacy can help minimize toxicities.

## Figures and Tables

**Table 1:** Summarized case studies assessed including author, sample size, and results.

Author	Sample Size	Results
Boden, R., et al.	169 (olanzapine/clozapine) 338 (other antipsychotics) 357,696 (unexposed)	Increased risk of Gestational diabetes (OR 1.77; 95% CI 1.04–3.03)
Kulkarni, J., et al.	147 (mothers)	142 live births, 25 pre-term (18%), 56 (43%) special care nursery, 20 (15%) with withdrawal, 8 (6%) congenital abnormalities. Most pregnancies had healthy babies
Ellfolk, M., et al.	1576 (FGA exposure) 4115 (SGA exposure) 22,125 (Unexposed)	Second Generation Antipsychotics (SGA) increased risk of Maternal diabetes (OR 1.43; 95% CI 1.25–1.65), C-section (OR 1.35; 95% CI 1.18–1.53), large for gestational age (LGA) (OR 1.57; 95% CI 1.14–2.16), and preterm birth (OR 1.29; 95% CI 1.03–1.62)
Auerbach, J.G., et al. [11]	29 (FGA exposure) 29 (Unexposed)	Mothers who take FGA during the third trimester have increased risk of having neonatal EPS
Habermann, F., et al. [14]	561 (SGA exposed) 284 (FGA exposed) 1122 (Unexposed)	Major malformation rates in the SGA cohort were higher than the unexposed cohort (OR: 2.17; 95% CI 1.20–3.91)
Cohen, L.S., et al.	214 (SGA) 89 (Unexposed)	Unlikely for SGA to increase the risk of major malformations (OR 1.25; 95% CI 0.13–12.19)
Diav-Citrin, O., et al.	188 (Haloperidol exposed) 27 (Penfluridol exposed) 631 (Unexposed)	Congenital abnormalities did not differ between cohorts (3.4% vs. 3.8%, *p* = 0.787). Higher rate of elective abortions (8.8% vs. 3.8%, *p* = 0.004), preterm birth (13.0% vs. 6.9%, *p* = 0.006), lower birth weight (3155 g vs. 3370 g, *p* < 0.001), and birth weight of full-term infants (3250 g vs. 3415 g, *p* = 0.004)
Cohen, L.S., et al.	4 prospective studies 12 case series 28 case reports Pharmaceutical reg.	Acceptable to feed: olanzapine, quetiapine Maybe acceptable: Haloperidol, chlorpromazine, risperidone, and zuclopenthixol Not enough data for other medications
Ilett, K.F., et al.	2 (mothers & infant)	No active metabolites of risperidone found in infant. Unlikely to be hazardous to breastfeeding individuals.
Peng et al.	76 (Antipsychotic exposure) 76 (Unexposed)	A statistically significant difference between cohorts at two months of age on BSID-III. No difference was noted at 12 months of age.
Garriga, M., et al.	23 (Antipsychotic resistant) 23 (Unexposed)	Birth weight (intrauterine environment) was a significant factor in predicting weight gain due to treatment(−7.4 +/− 3.2, *p* = 0.047)

Descriptions: first generation antipsychotics (FGA), second generation antipsychotics (SGA), large for gestational age (LGA).

**Table 2:** Adverse effects of antipsychotics that could be seen in the mother or infant.

	Possible Adverse Effects
Mother	Weight gain EPS Increased risk of maternal diabetes Increase rates of cesarean section
Fetus	Malformation, EPS, possibly as a form of neonatal abstinence or withdrawal Autonomic instability LGA Preterm Birth respiratory distress short-term developmental delay in infants in adaptive, motor, cognitive, and social-emotional behavior, but not in language, body weight, or height

Descriptions: extrapyramidal symptoms (EPS), large for gestational age (LGA).

## Data Availability

Data supporting the results above can be found on PubMed.
